# Large-scale fabrication of meta-axicon with circular polarization on CMOS platform

**DOI:** 10.1515/nanoph-2024-0413

**Published:** 2024-10-08

**Authors:** Gyu-Won Han, Jaewon Jang, Minsu Park, Hui Jae Cho, Jungchul Song, Yeonsang Park

**Affiliations:** Department of Physics, 26715 Chungnam National University, Daejeon, South Korea; Office of Nano Convergence Technology, National NanoFab Center, Daejeon, South Korea; 26715 Institute of Quantum Systems, Chungnam National University, Daejeon, South Korea

**Keywords:** metasurface, large-area fabrication, DUV photolithography, axicon, circular polarization

## Abstract

Metasurfaces, consisting of arrays of subwavelength structures, are lightweight and compact while being capable of implementing the functions of traditional bulky optical components. Furthermore, they have the potential to significantly improve complex optical systems in terms of space and cost, as they can simultaneously implement multiple functions. The wafer-scale mass production method based on the CMOS (complementary metal oxide semiconductor) process plays a crucial role in the modern semiconductor industry. This approach can also be applied to the production of metasurfaces, thereby accelerating the entry of metasurfaces into industrial applications. In this study, we demonstrated the mass production of large-area *meta*-axicons with a diameter of 2 mm on an 8-inch wafer using DUV (Deep Ultraviolet) photolithography. The proposed *meta*-axicon designed here is based on PB (Pancharatnam–Berry) phase and is engineered to simultaneously modulate the phase and polarization of light. In practice, the fabricated *meta*-axicon generated a circularly polarized Bessel beam with a depth of focus (DoF) of approximately 2.3 mm in the vicinity of 980 nm. We anticipate that the mass production of large-area *meta*-axicons on this CMOS platform can offer various advantages in optical communication, laser drilling, optical trapping, and tweezing applications.

## 1 Introduction

Metasurfaces, composed of subwavelength-scale structures, are thin, planar optical components capable of manipulating the amplitude, phase, and polarization of light [1]. The optical modulation characteristics of metasurfaces not only excel at reproducing or even surpassing the functions of conventional commercial optical components but also make metasurfaces highly attractive in various advanced fields such as drones [2], satellites [3], [4], [5], spectrometers [6], [7], [8], cameras [9], [10], [11], [12], holography [13], [14], [15], LiDAR [16], [17], vision [18] etc. Metasurfaces can be designed to exhibit various functionalities across different wavelength ranges, depending on the form of the structure and the materials used, and have been demonstrated in ultraviolet [19], [20], [21], [22], visible light [23], [24], [25], infrared [26], [27], [28], [29], terahertz [30], [31], and even acoustic regime [32] to date. Conventionally, high-resolution electron beam lithography (EBL) has been dominantly used to fabricate metasurfaces because of structure size in nanometer scale.

However, EBL is impractical for the mass production and large-area fabrication of metasurfaces due to the excessively long time required for drawing patterns. These drawbacks significantly limit the cost-effectiveness and versatility, posing challenges to the commercialization of metasurfaces. On the other hand, the patterning method based on photolithography with deep ultraviolet (DUV) or extreme ultraviolet (EUV) offers a significant advantage in that it allows for the large-scale drawing of metasurfaces, enabling efficient and rapid mass production. Therefore, in recent years, large-area metasurfaces and mass production of various metasurfaces based on lithography process have been demonstrated [33], [34], [35].

In 2020, Li et al. summarized and reported results on the large-scale fabrication of metasurfaces by a photolithography process until that time [36]. Complementary metal-oxide-semiconductor-compatible (CMOS-compatible) metasurfaces have been fabricated on Si or glass substrates, operating their functionality of lens, spectral filter, polarization bandpass filter (PBF), half wave plate (HWP), and beam deflector etc. in the range of visible to mid-infrared. In 2021, Tao et al. successfully demonstrated high-capacity optical wireless-broadcasting communications with beam-steering metasurfaces with the size of 2 mm × 2 mm fabricated on an 8-inch silicon-on-insulator (SOI) wafer by KrF lithography [37]. Additionally, ultraviolet, visible, and infrared metalenses have been manufactured by ArF immersion lithography and demonstrated in 2021, 2023, and 2024 [38]–[42]. These sequential results imply that metasurface manufacturing moves from research stage to mass-production stage, and almost approaches to real usage status. Among mass-manufactured metasurfaces, polarization-dependent components, which are a quarter-wave plate or half-wave plate, are as important as polarization-independent lens or beam deflector in the view of application [43], [44]. Especially, circular-polarized beam works important role in biosensing such as stimulated emission depletion microscopy (STED) [45]. Nevertheless, metasurfaces of Bessel beam with circular polarization property were not yet mass-manufactured by lithography. (See Supplementary Information 1)

**Figure 1:**
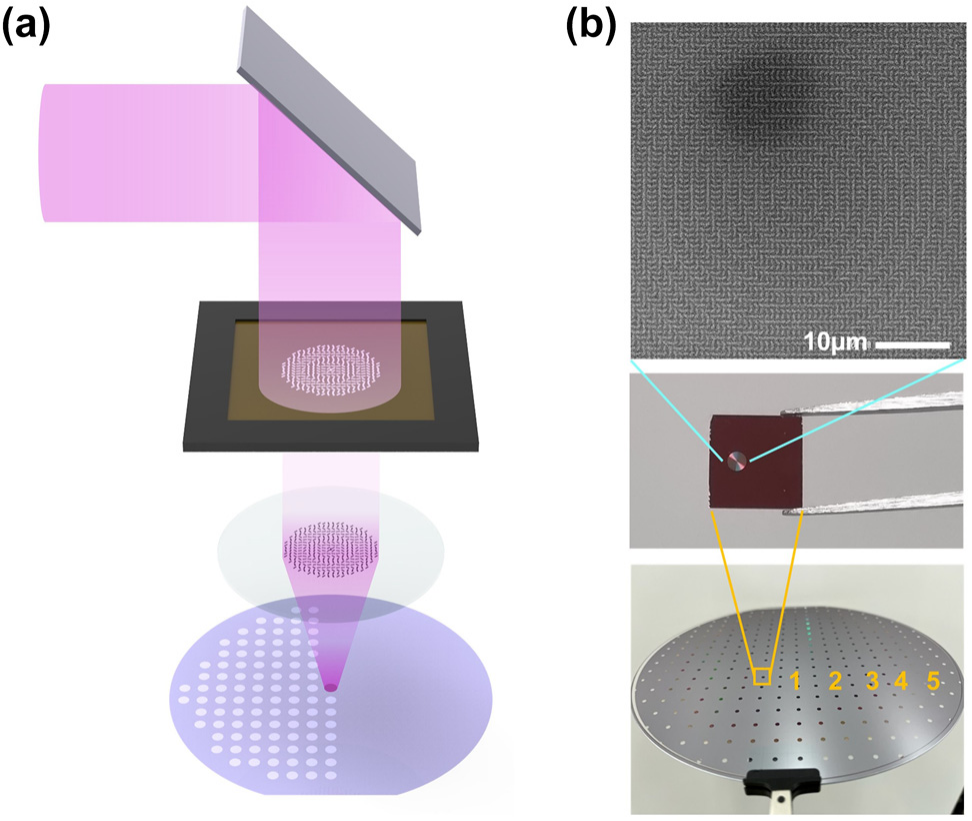
Large-scale fabrication of meta-axicons on a 8-inch wafer. (a) Schematic diagram of *meta*-axicon fabrication using DUV photolithography. (b) (Bottom) Photograph of 313 chips formed on an 8-inch glass wafer. The numbers 1 to 5 represent five samples selected from the middle to the outer edge of the wafer for uniformity verification. (Middle) Photograph of a single sample with a size of 2 mm. (Top) SEM image of fabricated *meta*-axicon sample.

Here, we demonstrated mass production of a metasurface-based axicon lens with a numerical aperture (NA) of 0.4, operating in the vicinity of 980 nm, using DUV photolithography, as shown in Figure 1(a). The *meta*-axicon is designed to form a Bessel beam with circular polarization based on geometric (Pancharatnam–Berry, PB) phase principles [46], [47], and was fabricated using a hydrogenated amorphous silicon (a-Si:H). Whole 313 meta-axicon chips with the diameter of 2 mm were mass-produced on an 8-inch quartz wafer, utilizing CMOS fabrication technology as shown in Figure 1(b). By measuring a beam shape, a depth of focus (DoF), and a polarization state of light transmitted through *meta*-axicons located at different positions on a wafer, we verified the uniformity of *meta*-axicons produced at the wafer scale. The fabricated samples showed ability of well-sustaining a Bessel beam shape within propagating length of about 2.3 mm, and clear circular polarization property. The introduction of well-established semiconductor production technologies such as DUV lithography to fabricate metasurfaces with circular polarization has been expected to open up new ways for the widespread application of metasurfaces across many domains because it is CMOS-compatible.

Recently, mass-manufacturing of metasurfaces with high efficiency using a nanoimprint lithography has been also reported by several research groups [48]–[52]. To make metasurfaces with high efficiency, to secure enough spatial room is essential, and it results into high cost in lithography process. Therefore, it is expected that manufacturing metasurfaces in a large scale with nanoimprinting technology can decrease cost drastically compared with the photolithography process. This approach gives us another route to mass-produce metasurface with high NA, and simultaneously a semiconductor lithography process is expected to still play a role in mass-production due to their fast speed and high reliability in manufacturing.

## 2 Design and simulation of *meta*-axicon

**Figure 2:**
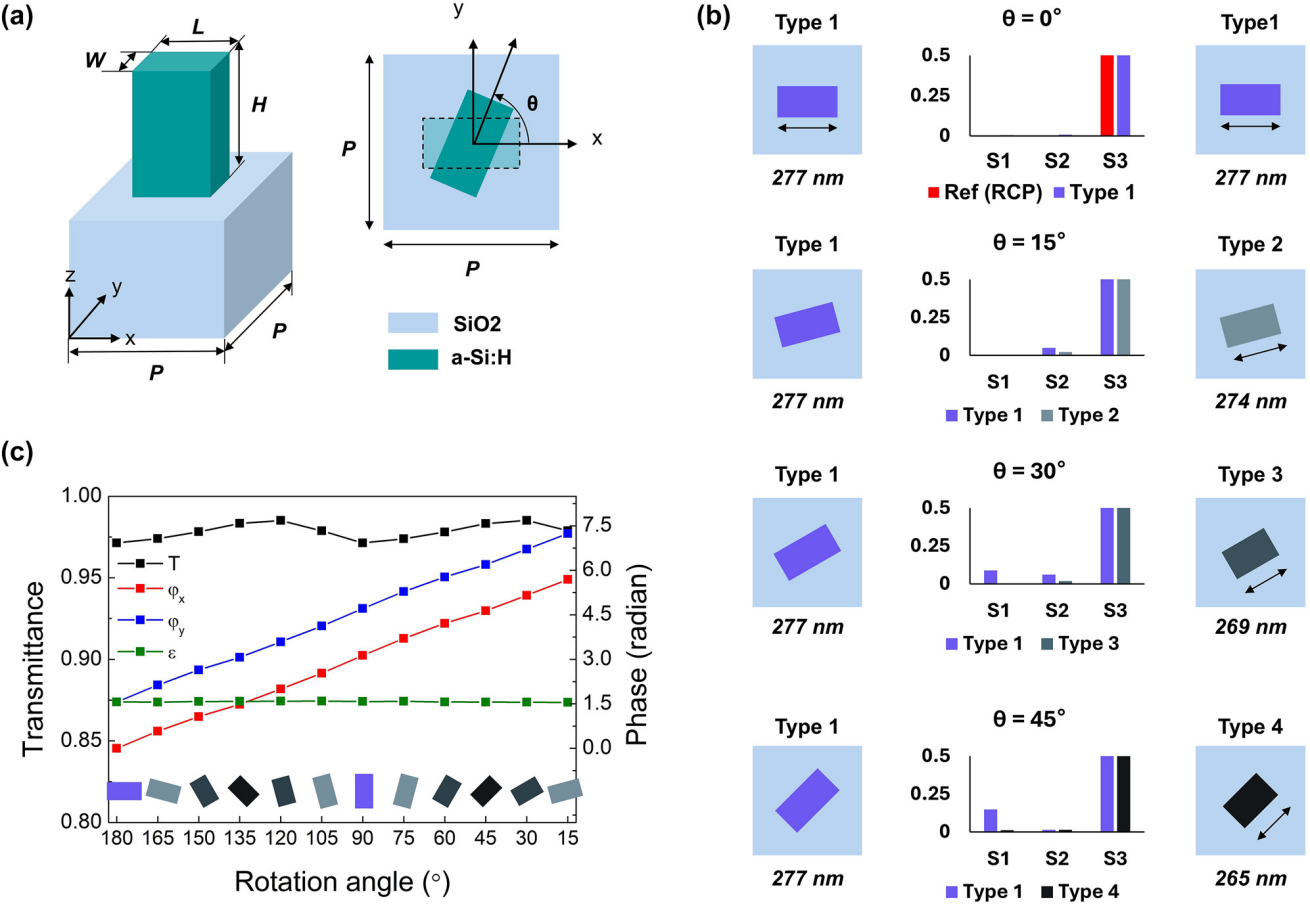
Design of the meta-atom structure. (a) Schematics of the meta-atom structure used in the *meta*-axicon design. A unit cell is composed of a-Si:H nanorod with a square lattice on a SiO_2_ substrate. (b) Diagram of Stokes parameter comparison between 4 types of meta-atom structure at the given rotation angle of *θ*. In diagram, the maximum range of Stokes parameter is limited as 0.5 for the magnification of *S*_1_ number and *S*_2_ number. Compared with value of Stokes parameters on type 1 in rotation angles of 15°, 30°, and 45°, values of Stokes parameters of type 2, type 3, and type 4 are closer to $\left(S_{1},S_{2},S_{3}\right)=\left(0,0,1\right)$ , which indicates perfect rightly-circularized polarization (RCP) state. (c) Graph presents calculated transmittance, phases of electric field in *x*- and *y*-direction (*φ*_*y*_ and *φ*_*x*_), and the phase difference between *φ*_*y*_ and *φ*_*x*_ (*ɛ*) as functions of the rotation angle *θ* when light with leftly-circularized polarization (LCP) state passes through the selected four unit cells.

A meta-atom can modulate the phase of the transmitted wave (written as *ϕ* in Equation (1)) from 0 to 2π solely through the rotation of its structure as shown in Figure 2(a), which is called as PB phase [53]. Simultaneously, when an anisotropic structure such as rectangle or ellipse is used as the meta-atom, each phase along each direction of anisotropy (written as *φ*_*x*_ and *φ*_*y*_ respectively in Equation (1)) can be also changed through transmission. If the phase difference (written as *ɛ* in equation (1)) between *φ*_*x*_ and *φ*_*y*_ is maintained as π/4 or π/2, the meta-atom with that anisotropic structure functions as a quarter-wave plate (QWP) or a half-wave plate (HWP) [43], [44]. Here, we have designed a *meta*-axicon with circular polarization by using the PB phase and anisotropic meta-atom. (See Supplementary Information 2)

(1) $$\begin{array}{cc} {\vec{E}}_{t} & ={\vec{E}}_{0}e^{\vec{i}\phi }=E_{x0}{\text{e}}^{\text{i}\varphi_{x}}\hat{x}+E_{y0}{\text{e}}^{\text{i}\varphi_{y}}\hat{y}\\ & ={\text{e}}^{\text{i}\varphi_{x}}\left(E_{x0}\hat{x}+E_{y0}{\text{e}}^{\text{i}\left(\varphi_{y}-\varphi_{x}\right)}\hat{y}\right)\\ & ={\text{e}}^{\text{i}\varphi_{x}}\left(E_{x0}\hat{x}+E_{y0}{\text{e}}^{\text{i}\varepsilon }\hat{y}\right)\;where\;\varepsilon =\varphi_{y}-\varphi_{x} \end{array}$$

To design a *meta*-axicon capable of forming a circularly polarized Bessel beam at a wavelength of 980 nm, we used hydrogenated amorphous silicon (a-Si:H, refractive index *n* = 3.74 @ *λ* = 980 nm) and glass (*n* = 1.45 @ *λ* = 980 nm) substrate because these materials are lossless at the target wavelength of 980 nm, and also very CMOS-compatible. A cuboid structure was used as a meta-atom and arranged in a square lattice with the period (*P*) of 375 nm to make *meta*-axicon as illustrated in Figure 2(a). The width (*W*) and the height (*H*) of the a-Si:H cuboid structure corresponded to 140 nm and 440 nm, respectively. However, we changed the length (*L*) of a meta-atom as four types with 265 nm, 269 nm, 274 nm, and 278 nm as shown in Figure 2(b) for the enhancement of polarization conversion efficiency. When rotating only one type of the cuboid meta-atom with a fixed square lattice, it imparts an effect similar to the deformation of the structure for incident light with fixed polarization. In other words, cuboids that are not rotated and those rotated by an arbitrary angle *θ* are not identical. This effective structural deformation caused by the rotation changes the phase difference of *ɛ*. This effective phase change gets higher as the rotation angle of *θ* increases. Therefore, when a metasurface that works as HWP and a axicon simultaneously is composed of only one type of meta-atom, it does not work perfectly and the efficiency of polarization conversion decreases because of a geometric phase variation.

To find out four types of meta-atom with different *L*, we used Stokes parameters $\left(S_{1},S_{2},S_{3}\right)$ shown in Equation (2) for transmitted waves. While changing *L* of a meta-atom with (*W*, *H*) = (140 nm, 440 nm) and rotating the angle *θ* with given *L*, we calculated Stokes parameters of the transmitted waves and compared them with $\left(S_{1},S_{2},S_{3}\right)=\left(0,0,1\right)$ , which corresponds to Stokes parameters of perfect right-hand circular polarization (RCP). Figure 2(b) presents Stokes parameters calculated in meta-atoms with different *L* named as type 2, 3, and 4 while comparing with Stokes parameters calculated in the meta-atom named as type 1. As shown in Figure 2(b), as the rotation angle of *θ* increases, we can see that the values of $\left(S_{1},S_{2}\right)$ in type 1 meta-atom with *L* of 277 nm deviates from zero. On the contrary, type 2, 3, and 4 meta-atoms with *L* of 274 nm, 269 nm and 265 nm respectively presented in the right column of Figure 2(b) show almost zero value at the given angle *θ*. By reducing *L* of each meta-atom as *θ* increases, it seems that we can compensate the structural deformation of anisotropy. We used type 1, 2, 3, and 4 meta-atoms with *L* of 277 nm, 274 nm, 269 nm, and 265 nm respectively as the basic building blocks consisting of a metasurface with circular polarization.

(2) $$\begin{array}{cc} S_{0} & =\left\langle E_{x0}^{2}\right\rangle +\left\langle E_{y0}^{2}\right\rangle \\ S_{1} & =\left\langle E_{x0}^{2}\right\rangle -\left\langle E_{y0}^{2}\right\rangle \\ S_{2} & =\left\langle 2E_{x0}E_{y0}\cos\varepsilon \right\rangle \\ S_{3} & =\left\langle 2E_{x0}E_{y0}\sin\varepsilon \right\rangle \end{array}$$

Figure 2(c) presents calculated transmittance, *φ*_*x*_, *φ*_*y*_, and *ɛ* of 12 unit cells forming a *meta*-axicon with circular polarization at the wavelength of 980 nm. Four types of meta-atom were indicated as different colors and drawn in function of rotation angle *θ* with respect to *x*-direction. From Figure 2(c), we can see that all unit cells maintain a transmittance of over 97 %, and the phase of transmitted wave increases linearly from 0 to 2π while maintaining the phase difference of *ɛ* as π/2.

**Figure 3:**
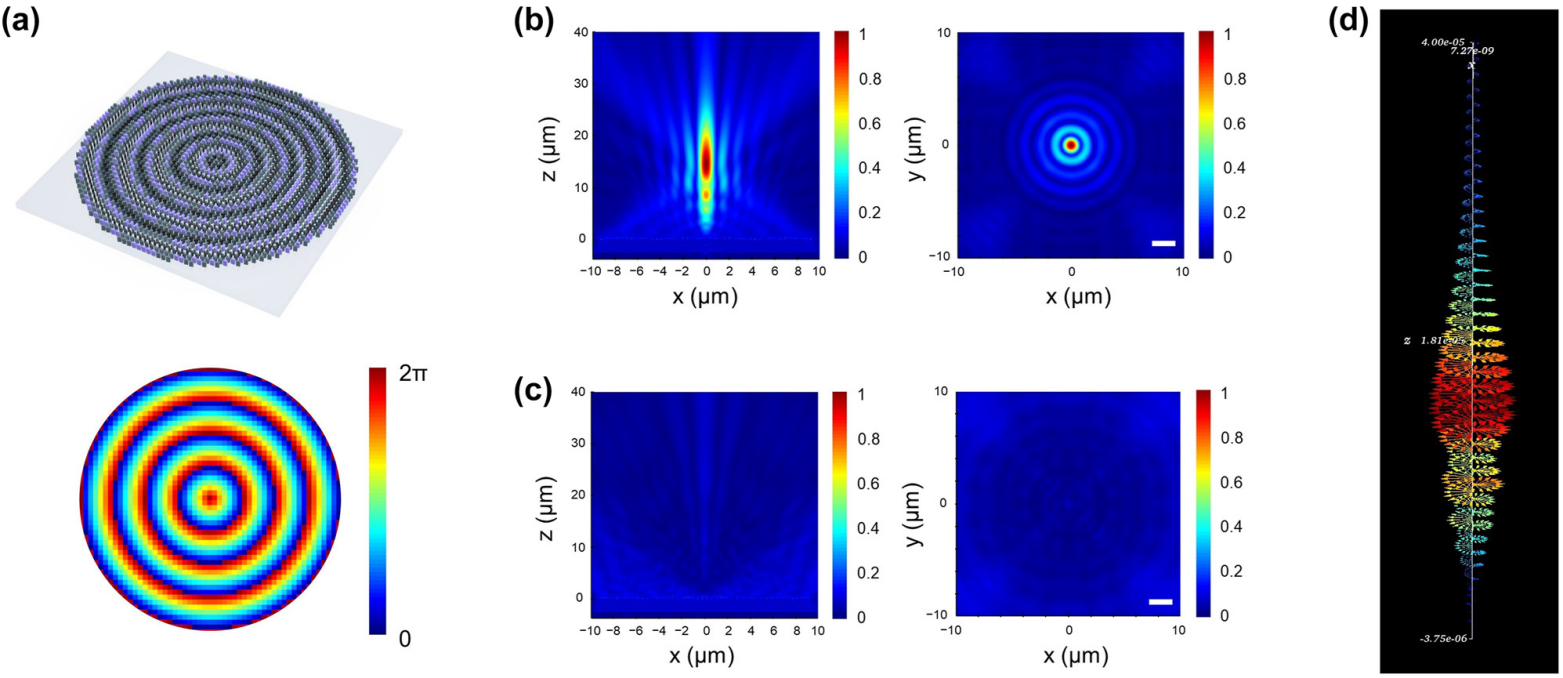
Design and simulation results of the *meta*-axicon. (a) (Top) schematic of a *meta*-axicon with a diameter of 20 µm and NA of 0.4 composed of four-type meta-atom structures. (Bottom) Phase distribution map of an axicon according to Equation (3). (b) Simulated electric field (E-field) profiles with rightly-circulated polarization (RCP) state transmitted through the *meta*-axicon at the wavelength of 980 nm. E-field profile in *xz*-plane (left) shows well-aligned Bessel beams, while E-field profile in *xy*-plane (right) exhibits intensity distribution resembling the first-kind zero-order Bessel function. (c) Simulated E-field profiles with leftly-circulated polarization (LCP) state transmitted through the *meta*-axicon at the wavelength of 980 nm. E-field profiles in *xz*- (left) and *xy*-plane (right) show a beam spreading and disappearance. (d) Vector plot of the E-field along the propagation axis of the Bessel beam shown in (b), indicating the formation of well-defined circular polarization states.

Figure 3(a) shows schematic and a phase map of *meta*-axicon that was calculated from Equation (3) [54]. Using finite-difference time-domain simulator (Lumerical Inc.), electric field (E-field) distributions of transmitted wave were calculated in 3-dimensionally when left-hand circularly polarized (LCP) planewave is incident to the *meta*-axicon with a NA of 0.4 and a diameter of 20 μm designed at the target wavelength of 980 nm.

(3) $$\begin{array}{c} \varphi \left(x,y\right)=2\pi -\frac{2\pi }{\lambda_{d}}\cdot \sqrt{x^{2}+y^{2}}\cdot NA\\ DoF=\frac{D}{2\tan\left(\sin^{-1}\left(NA\right)\right)} \end{array}$$

From Figure 3(b), we can see that the transmitted waves with RCP state are well collimated in the propagation direction of *z*, and their E-field map in the *xy* plane forms a distribution of the first-kind zero-order Bessel function *J*_0_. From E-field calculated in the *xz* plane, the DoF was obtained as about 23 μm, and it was in good agreement with the theoretical value of 22.92 μm derived from equation (3) within an error range of 7 %. On the contrary, when plane waves with the RCP state are incident into the *meta*-axicon, transmitted waves with the LCP state converted by *meta*-axicon spread outwards on both sides as shown in Figure 3(c).

Additionally, to make sure of forming Bessel beam with circular polarization conversion via the *meta*-axicon, we represented E-field along the propagating axis of the collimated beam in vector form as shown in Figure 3(d). This allows us to visually confirm that the transmitted beam has a circularly polarized state. By comparing the Stokes parameter values of the incident and transmitted waves, the handedness conversion from LCP to RCP could be verified again. The initial Stokes parameter values for the incident light were $\left(S_{1},S_{2},S_{3}\right)=\left(0,0,-1\right)$ , which means a perfect LCP state, and the calculated values for the transmitted waves after passing through the *meta*-axicon were $\left(S_{1},S_{2},S_{3}\right)=\left(0.0259,-0.0056,0.9997\right)$ . Thus, we could confirm that the incident LCP light was converted into RCP light well.

## 3 Fabrication of *meta*-axicon

**Figure 4:**
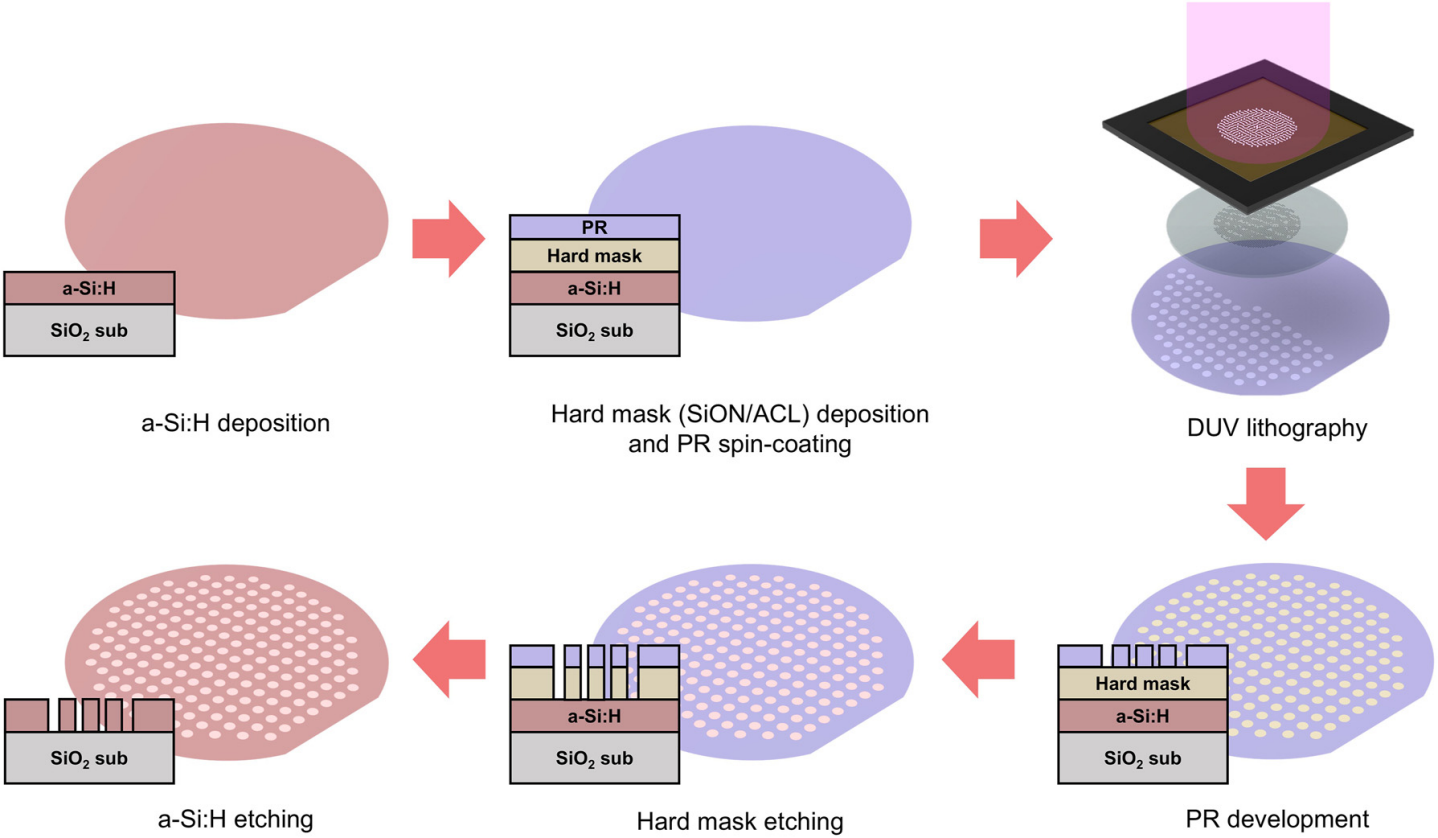
Schematics of whole processes fabricating *meta*-axicon using DUV lithography.

Figure 4 illustrates the entire fabrication process of the *meta*-axicon. We used a transparent 8-inch glass wafer as the substrate at the target wavelength of 980 nm, onto which a 440 nm-thick a-Si:H layer was deposited via PECVD (plasma enhanced chemical vapor deposition). Subsequently, to ensure that the process equipment sensors can recognize the pure glass wafer, an opaque 200 nm-thick layer of titanium (Ti) was deposited as a chucking material on the backside of the glass wafer. Then, hard masks consisting of a 500 nm-thick amorphous carbon layer (ACL) and a 30 nm-thick silicon oxynitride (SiON) layer were deposited using PECVD. This combination of hard mask layers improved the etching margin and controlled reflection, resulting in clearer patterns with a higher aspect ratio. Following this, a positive-type photoresist (PR) was spin-coated with a thickness of 110 nm. Subsequently, patterning was performed on the wafer using an ArF dry scanner with a 193 nm excimer laser as the light source (ASML XT1250D). The *meta*-axicon patterns were printed on the wafer at a four-fold reduced size through the optical system, and then the PR was developed. When executing a lithography, we adjusted very small size difference between neighboring meta-atoms finely by calibrating an exposure energy precisely before the patterning of actual samples and biasing an exposure energy gradually according to the size of nanorods. Next, a dry etching process was conducted to transfer the developed PR patterns to the hard mask and sequentially a-Si:H layer. The ACL and SiON hard mask layers were etched using a capacitively coupled plasma (CCP) etcher, followed by etching of the a-Si:H layer using an inductively coupled plasma (ICP) etcher. Finally, the remaining PR was removed using O_2_ plasma. As shown in Figure 1(b), total 313 samples of *meta*-axicon with a diameter of 2 mm were mass-produced on a only single 8 inch wafer.

After etching process, we measured widths and lengths of nanorods located at different positions on a single 8-inch wafer to check out the fabrication error of mass-producing metasurfaces. From scanning electron microscope (SEM) images of five samples, we could obtain average fabrication error of 10 % in width and 2 % in length. (See Supplementary Information 3)

## 4 Experimental results

### 4.1 Imaging of Bessel beam

**Figure 5:**
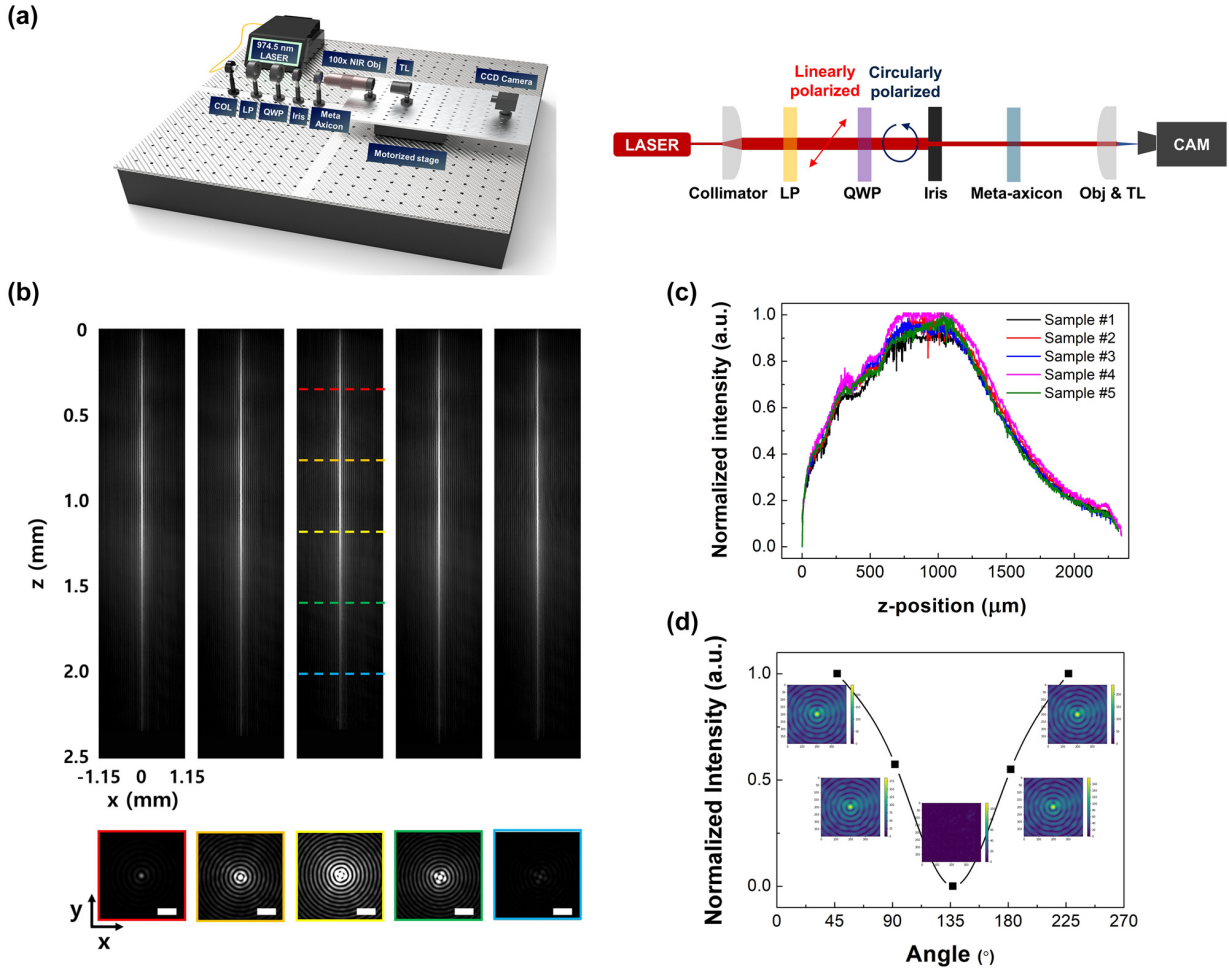
Measurement of the fabricated meta-axicons. (a) Schematic of the optical imaging measurement setup. COL, fiber collimator; LP, linear polarizer; QWP, quarter wave plate; TL, tube lens. (b) (Top) Intensity distribution images in the *xz* cross-section measured in the transmission direction for five samples. (Bottom) Intensity distribution images in the *xy* cross-section measured in the regions indicated by colored dashed lines in *xz* cross-section. (c) Plot of the intensity measured for the five samples as a function of the transmission direction. (d) Graph of intensity distribution as a function of the QWP rotation angle, with the inset displaying images captured by the CCD camera.

To verify the wavefront modulation capability of the fabricated *meta*-axicon, we first conducted beam imaging experiments. Simultaneously, identical experiments were executed on five samples obtained from the center to the periphery of the 8inch wafer to validate the uniformity of the samples produced on a wafer scale. Measurements were performed using the optical imaging setup shown in Figure 5(a). A laser diode with a central wavelength of 974.5 nm (PL-FP-974-A-A81-SA-FBG, LD-PD Inc.) was used as the light source, which was transformed into a collimated beam with a beam size of 4 mm by use of a fiber collimator. The output laser beam was modulated to the LCP state through a subsequent linear polarizer and a QWP, and then resized to 2 mm beam size through an iris before being incident on the fabricated *meta*-axicon to match the beam size and the sample size. To see the form of the generated beam, a 100× NIR objective lens, a tube lens, and a charge coupled device (CCD) camera were located behind the sample. Imaging components are arranged on a motorized stage, and the position was swept in the propagation direction of *z* with the help of a motion controller to collect image data for each position and integrate them into one image. As a result, it was observed that the beam transmitted through the *meta*-axicon, as shown in Figure 5(b), exhibited a well-collimated Bessel beam shape. From obtained images, we could confirm that the intensity distribution along the beam cross-section was found to follow the distribution of a first-kind zero-order Bessel function.

To find out the DoF of fabricated *meta*-axicon, the measured beam intensities for samples 1 to 5 were drawn as a function of *z*-position in the direction of wave propagation as shown in Figure 5(c). All five samples exhibit nearly identical intensity distributions, confirming the excellent quality of the samples fabricated at the wafer scale. Table 1 presents the DoF values obtained for samples 1 to 5 from Figure 5(c). Whole results were closely matched to the theoretical value of 2.2913 mm calculated using Equation (3) for an NA of 0.4 and a diameter of 2 mm for the axicon, within a 2.5 % error margin. (See Supplementary Information 4) By comparing the forementioned error in fabrication and DoF values measured from 5 different samples, we could conclude that fabrication error in length affected the performance of device by almost similar amount. (See Supplementary Information 3)

**Table 1:** Depth of focus (DoF) values measured for five different samples indicated in Figure 1(b).

Sample	1	2	3	4	5
DoF (mm)	2.2747	2.3027	2.2640	2.3453	2.3240

### 4.2 Measurement of circular polarization

The meta-atoms composing the *meta*-axicon are anisotropic, and hence the wavefront of transmitted light inevitably depends on the polarization state of the incident light. In the case of the presented samples, when LCP light is incident, it converts to RCP light, forming a Bessel beam, whereas when RCP light is incident, it is designed to convert to LCP light, spreading outward. Similarly, when a linearly polarized (LP) light, which can be considered as a 1:1 linear combination of RCP and LCP light, is incident, it forms an RCP state Bessel beam with half the intensity of the case when LCP light is incident. To quantitatively confirm this, we controlled the polarization state of light incident on the *meta*-axicon by rotating a QWP in the setup and measured the photon counts detected by the CCD. Intensities at the rotation angle were calculated by integrating whole counts taken by CCD in the setup, and they were normalized with background signal of the CCD. Obtained data were plotted as the function of the rotation angle of the QWP as shown in Figure 5(d). Insets in Figure 5(d) correspond to images where intensity is calculated respectively. From the plotted graph, we could see sinusoidal polarization dependence on the rotation angle clearly, and make sure that fabricated *meta*-axicon has stable polarization conversion capability.

## 5 Conclusions

In conclusion, we designed a metasurface-based axicon with an NA of 0.4 operating at a central wavelength of 980 nm, and demonstrated mass-production of 313 chips with a diameter of 2 mm on an 8 inch wafer using CMOS-compatible processes. The produced *meta*-axicon depends on the polarization state of the incident light, forming a Bessel beam in the RCP state when illuminated by LCP plane waves. Additionally, we confirmed the formation of a well-collimated first-kind zero-order Bessel beam through beam imaging experiments, and further investigated the dependence on the polarization of the incident light by modulating the polarization. To confirm the uniformity of the mass-produced samples on the wafer, we selected five samples located from the center to the edge of the wafer among 313 chips and repeated the same measurements.

Through measurement of the axial intensity distribution of the Bessel beam, we confirmed that the *meta*-axicon produced on the wafer accurately reproduces the intensity distribution of the actual axicon transmission pattern owing to an ArF dry stepper process that already have been used in mass-production. Additionally, the measured DoF values closely matched the theoretical values of 2.29 mm within a 2.5 % margin of error. Based on these findings, we expect that the mass production of thin and flat *meta*-axicons on the CMOS platform will not only offer cost advantages but also numerous benefits such as relatively flexible NA design capabilities, polarization modulation capabilities, optical tweezing and trapping, high precision laser processing applications, and beyond.

## Supplementary Material

Supplementary Material Details
