# Correction to “Large‐Scale Fabrication of Meta‐Axicon With Circular Polarization on CMOS Platform”

**DOI:** 10.1002/nap2.70127

**Published:** 2026-05-09

G.‐W. Han, J. Jang, M. Park, H. J. Cho, J. Song, and Y. Park, “Large‐Scale Fabrication of Meta‐Axicon With Circular Polarization on CMOS Platform,” *Nanophotonics* 13 (2024): 4337–4345, https://doi.org/10.1515/nanoph‐2024‐0413.

The Acknowledgments section contained an error in the year specified for the BK21 FOUR Program funding, which is herewith corrected from 2022 to 2024. The correct Acknowledgment section is given below.

This study was supported by a research fund from Chungnam National University, the National Research Foundation of Korea Grant funded by the Korean Government (MOE), the BK21 FOUR Program by the Chungnam National University Research Grant, 2024, the Basic Science Research Program through the National Research Foundation of Korea (NRF) funded by the Ministry of Education (NRF‐2020R1A6A1A03047771), and the Technology Innovation Program (or Industrial Strategic Technology Development Program) (“RS‐2023‐00235080”, Development of layered color filters for enhancing low light sensitivity) funded By the Ministry of Trade, Industry & Energy (MOTIE, Korea) (1415187384).

We apologize for this error.

