# Supplementary material for: Large-scale fabrication of meta-axicon with circular polarization on CMOS platform
Source: Nanophotonics. 2024 Oct 8;13(23):4337–45. doi: 10.1515/nanoph-2024-0413 (PMC11636471; doi:10.1515/nanoph-2024-0413)
Supplement: Supplementary file 1 — Supplementary Material Details [file j_nanoph-2024-0413_suppl_001.docx]

**Supplementary Information**

**Large-Scale Fabrication of Meta-Axicon with Circular Polarization on CMOS Platform**

Gyu-Won Han^1,3^ †, Jaewon Jang^1,2^ †, Minsu Park^1,2^, Hui Jae Cho^3^, Jungchul Song^3^*, and Yeonsang Park^1,2^*

*^1^Departement of Physics, Chungnam National University, Daejeon, Korea*

*^2^Instutte of Quantum Systems, Chungnam National University, Deajeon, Korea*

*^3^Office of Nano Convergence Technology, National NanoFab Center, Deajeon, Korea*

*† These authors contributed equally to this work.*

** E-mail:* [*yeonsang.park@cnu.ac.kr*](mailto:yeonsang.park@cnu.ac.kr)*,* [*justsong@nnfc.re.kr*](mailto:justsong@nnfc.re.kr)

**S1. Summary of large-scale fabrication of metasurfaces**

We summarized various results on the large-scale fabrication of metasurfaces in Table S1. The former parts of Table S1 are same to the table in ref. 36. In the latter parts of Table S1, we arranged recent results on mass-production of metasurface using lithography and nanoimprinting technology. From Table S1, it can be seen that manufacturing metasurfaces forming Bessel beams with circular polarization in large-scale was not reported yet. It is expected that the demonstration of metasurfaces with circular polarization on the CMOS platform in a large-scale will raise the possibility of applying metasurfaces into industry in polarization control as well as beam shaping of light.

**Table S1**: Summary of recent results on manufacturing metasurface on a CMOS platform.

| **Fabrication approach** | **Meta-surface**  **materials** | **substrate** | **Functional device** | **Lithography**  **tool** | **Meta-atom structure; size; thickness** | **wavelength** | Reference (year) |
| --- | --- | --- | --- | --- | --- | --- | --- |
| Direct etching on Si wafer | c-Si | Si wafer | PBF | 193 nm immersion scanner | Pillar; 156 nm; 750 nm | Near-IR  (1310 and 1610 nm) | Opt. Express 27, 26060-26069 (2019) |
|  |  |  | HWP |  | Pillar 180 nm;1700 nm | Near-IR  (1700 nm) | Nanophotonics 9, 149-157 (2019) |
|  |  |  | Lens | UV lithography | Pillar; 1500 nm;6800 nm | Mid-IR  (10,600 nm) | Applied Physics Letters 113.20 (2018) |
|  |  |  | Microlens array |  |  |  | Opt. Express 27, 10738-10744 (2019) |
| Pattering on top of the dielectric layer | a-Si | SiN on Si wafer | Spectral filter for color display | 193 nm immersion scanner | Pillar; 70 nm; 130 nm | Visible  (400-800 nm) | Opt. Express 26, 19548-19554 (2018) |
|  |  | SiO2 on Si wafer | Lens |  | Pillar; 100 nm; 850 nm | Near-IR  (1550 nm) | 2019 IEEE 16th International Conference on Group IV Photonics (GFP), vol.1949-209X, pp. 1-2 (2019) |
| Layer transfer onto glass wafer |  | Glass wafer | Beam deflector  array |  | Pillar; 221 nm; 100 nm | Near-IR  (940 nm) | Nanophotonics 8, 1855-1861 (2019) |
|  |  |  | lens |  | Pillar; 100 nm; 600 nm | Near-IR  (940 nm) | Nanophotonics 9, 823-830 (2020) |
|  |  |  | Spectral filter |  | Pillar; 130 nm; 400 nm | Visible  (400-800 nm) | Conference on Lasers and Electro-Optics*,* p. STh1O.4 (2019) |
| Layer transfer onto stretchable electrodes |  | Carbon nanotube (stretchable electrodes) | Lens | 365 nm stepper | Pillar; 810 nm; 950 nm | Near-IR  (1550 nm) | Science advances 4, eaap9957 (2018) |
| Patterning on glass wafer | a-Si | Glass wafer | Lens | 365 nm stepper | Pillar; 830 nm;600 nm | Near-IR  (1550 nm) | Opt. Express 26, 1573-1585 (2018) |
|  |  |  |  | 193 nm immersion scanner | Pillar; 100 nm; 400 nm | Near-IR  (940 nm) | Optical Fiber Communication Conference, p. Th2A. 8. (2020) |
|  | SiO2 |  |  | 248 nm stepper | Tapered pillar; 250 nm; 1500 nm | Visible  (400-700 nm) | Nano Letters 19, 8673-8682 (2019) |
| Patterning on Si or glass wafer | SiN | Si and quartz substrates | Lens | 365 nm stepper | Pillar; 500 nm; 1500 nm | Visible  (633 nm)  Near-IR  (1550 nm) | Optica 5, 825-831 (2018) |
| Patterning on glass wafer or SiO2 wafer | Au | SiO2 on Au film and glass wafer | Lens | 365 nm stepper | Disc; 800 nm; 50 nm | Mid-IR  (4600 nm) | Opt. Express 24, 18024-18034 (2016) |
|  |  | SiO2 on Au film and Si membrane |  |  |  |  | APL Photonics 3, 021302 (2018) |
| Patterning of W on SiO2 | W | SiO2 on W and glass substrate | Thermal emitter |  | Disc; 350 nm; 50 nm | Visible to mid-IR  (600-2000 nm) | Micromachines 10, 157 (2019) |
| Lift-off process | Au and insulator (Si/SiO2) | Si substrate | Thermal emitter and absorber |  | Disc; 550 nm; 50 nm | Mid-IR | Scientific Reports 9, 8284 (2019). |
| Patterning on top of the dielectric layer | c-Si | SOI wafer | Beam steerer | 248 nm DUV lithography | Pillar; 300 nm, 340 nm | Near-IR  (1550 nm) | Advanced Materials 34, 2106080 (2022) |
| Patterning on top of the dielectric or metal layer and DRIE etching | Ge, Al | Al2O3 | Mid-IR photonics and biosensing | 248 nm stepper | Pillar; 300 nm; 340 nm | Mid-IR  (3450 nm, 6250 nm, 6500 nm) | Advanced Materials 33, 2102232 (2021) |
| Pattering on Si wafer and nanoimprint lithography | Resin with TiO2 | SiO2 | Lens | 193 nm immersion scanner | Pillar; 70 nm; 900 nm | Visible  (400-700 nm) | Nature Materials 22, 474 481 (2023) |
| Pattering on top of the dielectric layer and nanoimprint lithography | Resin with ZrO2 | SiO2 | Lens | 193 nm immersion scanner | Pillar; 60 nm; 550 nm | Ultraviolet  (325 nm) | Materials Today 73, 9-15 (2024) |
| Nanoimprint lithography | a-Si:H or resin with Si | Glass wafer | Lens | 193 nm dry scanner | Pillar; ~ 170 nm; 700 nm  Pillar; ~150 nm; 700 nm | Near-IR  (940 nm) | Laser & Photonics Reviews 18, 2300929 (2024) |
|  | Nano-ink  (HPC with TiO2) | Various substrates | Water-soluble food label | 193 nm immersion scanner | Pillar; 190 nm; 300 nm | Visible  (400-700 nm) | Nature Food 5, 293-300 (2024) |
|  | Resin with TiO2 |  | Hologram |  | Pillar; 116 nm, 940 nm | Visible  (400-700 nm) | Laser & Photonics Reviews 16, 2200098 (2022) |
|  | Resin with ZrO2 |  |  |  | Pillar; 65 nm; 700 nm  Pillar; 45 nm; 700 nm | Near-UV to Deep-UV  (325 nm, 248 nm) | Light: Science & Applications 12, 68 (2023) |
|  | Resin with TiO2 | Glass substrate |  |  | Pillar; 120 nm; 910 nm | Visible  (400-700 nm) | Advanced Materials 35, 2208520 (2023) |
|  |  | Top of the cell |  |  | Pillar; 120 nm; 940 nm | Visible  (400-720 nm) | Advanced Materials 36, 2311785 (2024) |
|  |  | Various substrates | Lens | 193 nm immersion lithography | Pillar; 120 nm; 700 nm | Visible  (400-700 nm) | PhotoniX, 4, 18 (2023) |
|  | SiN | Fused silica | Lens |  | Pillar; 100 nm; 650 nm | Visible  (500 nm) | Advanced Optical Materials 12, 2301562, (2024) |

**S2. Design of meta-atom for the circular polarization**

When designing meta-atoms under a square lattice and periodic boundary conditions, if a rectangular meta-atom designed as a Half-Wave Plate (HWP) at $\theta$= 0° is rotated by an arbitrary angle $\theta$ on the same square lattice and periodicity, the optical response of the system is no longer perfectly identical for the initial incident light polarization state (in this case, LCP consisting of x and y polarizations with a 90° phase delay). This ultimately impairs the polarization conversion efficiency of the meta-atom designed as an HWP.

For instance, consider the situation depicted in Figure S2(a), where a rectangular structure is periodically arranged at 0° with respect to the x-axis in a square lattice, and compare it to the structure in Figure S2(b), where the rectangles are periodically arranged with a 45° rotation. If the left case in Figure S2(a) ($\theta$ = 0°) is designed as a perfect HWP, then the entire structure will still function effectively as an HWP even when rotated by an arbitrary angle (as shown on the right in Figure S2(a)). In contrast, if the same-sized rectangular structures are rotated by an arbitrary angle at the unit cell level and then arranged periodically (for example, $\theta$ = 45°) as shown on the left in Figure S2(b), this situation differs from that in Figure S2(a). In this case, the arrangement of adjacent meta-atoms changes, resulting in different interactions between meta-atoms compared to the arrangement in Figure S2(a). Therefore, this arrangement will not function as a perfect HWP.

Figure S2(c) shows the calculated phase difference between $\varphi_{y}$ and $\varphi_{x}$ ($\varepsilon),$as functions of the rotation angle $\theta$. This calculation was additionally performed for a single meta-atom (Type 1, L = 277 nm) designed as an HWP at $\theta$ = 0° (black line), with the meta-atom rotated in 15° decrements. For comparison with the optimized case mentioned in the main text, the phase difference values from Figure 2(c) are also shown with a red line. As can be seen, the phase difference between the two electric field components is relatively less uniform when only Type 1 is used.

Here, to correct for the effects of rotation in the rectangular structure, we calculated the polarization conversion efficiency (PCE) of the meta-atoms with respect to $\theta$ and L, as shown in Figure S2(d). The angle was plotted with a 5° interval, and L was plotted with a 1 nm interval. A value of 1 on the scale bar indicates that the polarization state of the transmitted wave is perfect circular polarization, which means 100% polarization conversion efficiency. Based on these calculations, we selected four different types of unit cells for the design of the meta-axicon, which are marked with differently colored circles in Figure S2(d) corresponding to the colors of types 1 through 4 in the main text.


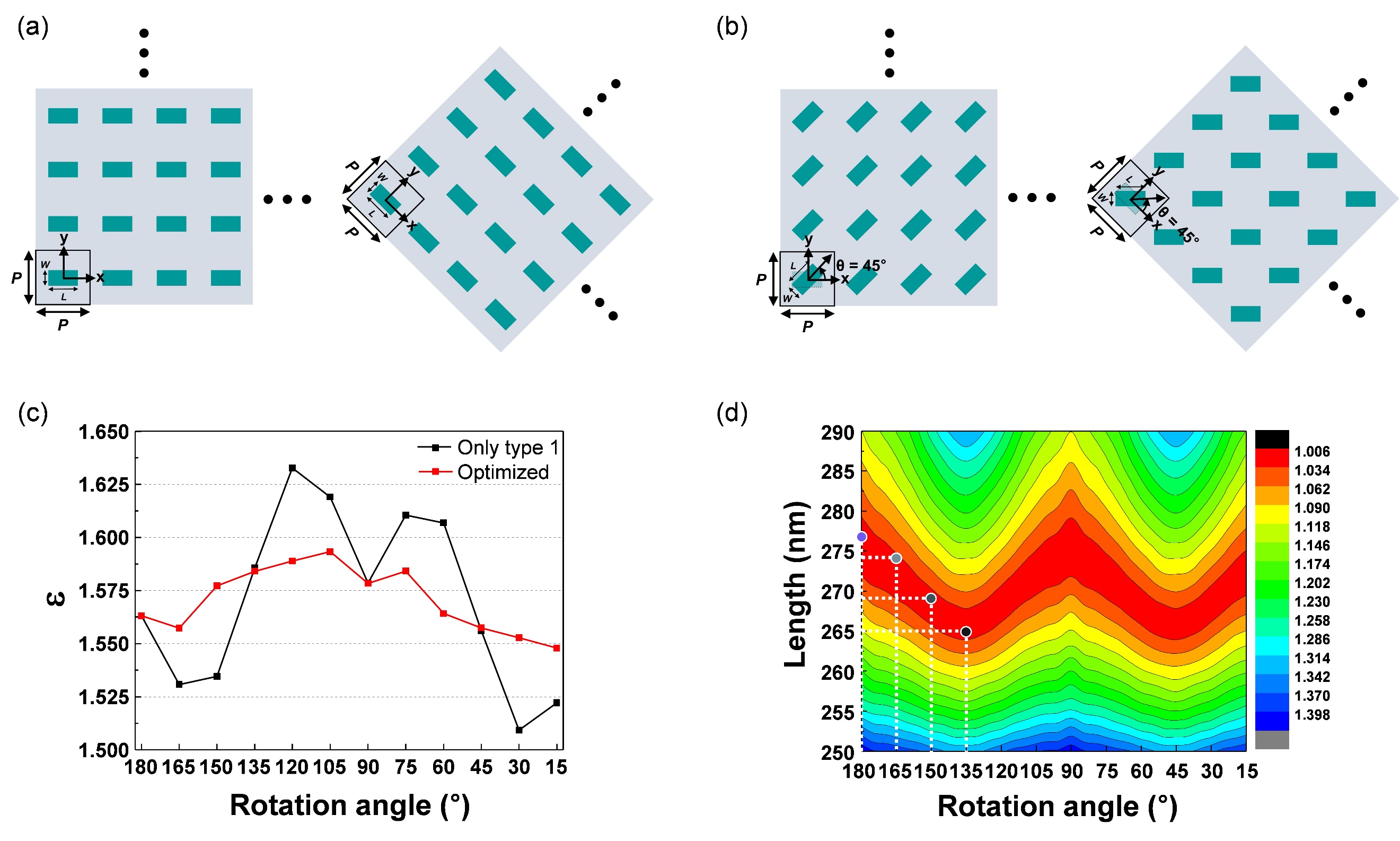


**Figure S2:** (a) A perfect HWP metasurface composed of a periodic array of anisotropic meta-atoms aligned in the x-direction (θ = 0°) in a square lattice with period P (left), and the same structure rotated by an arbitrary angle (right). (b) A structure composed of a periodic array of meta-atoms rotated by 45 degrees with respect to the x-axis in a square lattice (left), and the same structure rotated by an arbitrary angle (right). (c) Phase difference (ε) of the two electric field components as a function of the meta-atom rotation angle θ. The black line represents the case using only type 1 structures, while the red line shows the optimized result using a combination of types 1 to 4. (d) Polarization conversion efficiency map as a function of rotation angle θ and L, where a value of 1 represents 100% polarization conversion efficiency. The differently colored circles represent meta-atoms of types 1 to 4, with colors corresponding to those shown in the main text.

**S3. Analysis of the fabrication error**

To find out the fabrication error, we measured length and width of five nanorods located at different position in an 8-inch wafer as shown in Fig. S3(a). From SEM images in Fig. S3(b), we could obtain average fabrication error $\left( error=\frac{x_{meas}-x_{target}}{x_{target}} \right)$ of 10 % in width and 2 % in length. By comparing fabrication error measured from Fig. S3(b) and DoF values of 11 samples shown in Table S2, we could guess that fabrication error within 10 % affects the device performance little because average DoF values obtained from 11 different samples are 2.320 mm almost similar with the theoretical value of 2.291 mm and their errors are in the range of about 2.0 %.


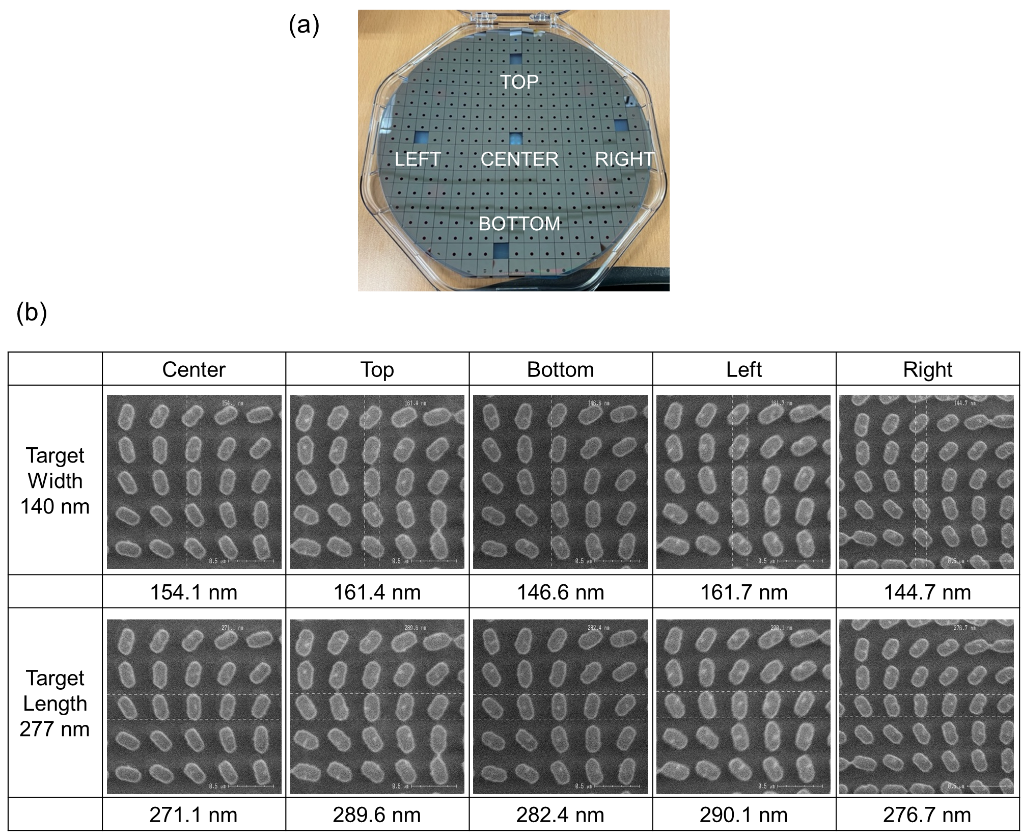


**Figure S3.** (a) Positions of 5 samples selected for checking out the fabrication error. (b) Scanning electron microscope (SEM) images of five samples selected at the position shown in (a).

**S4.** **Additional verification of wafer-scale uniformity**

To assess the uniformity of meta-axicons fabricated at the wafer scale, Depth of Focus measurements were additionally performed using a photodiode sensor, differing from the beam imaging method described in the main text. The numbers on Figure S4 indicate the positions of the samples used in these measurements. Samples numbered 1 through 5 correspond to the same samples measured using the beam imaging method, while samples numbered 6 through 11 were selected along the diagonal direction from the center of the wafer. The measured DoF values in both directions show uniformity within approximately 2.08% of the theoretical value of 2.2913 mm.

**
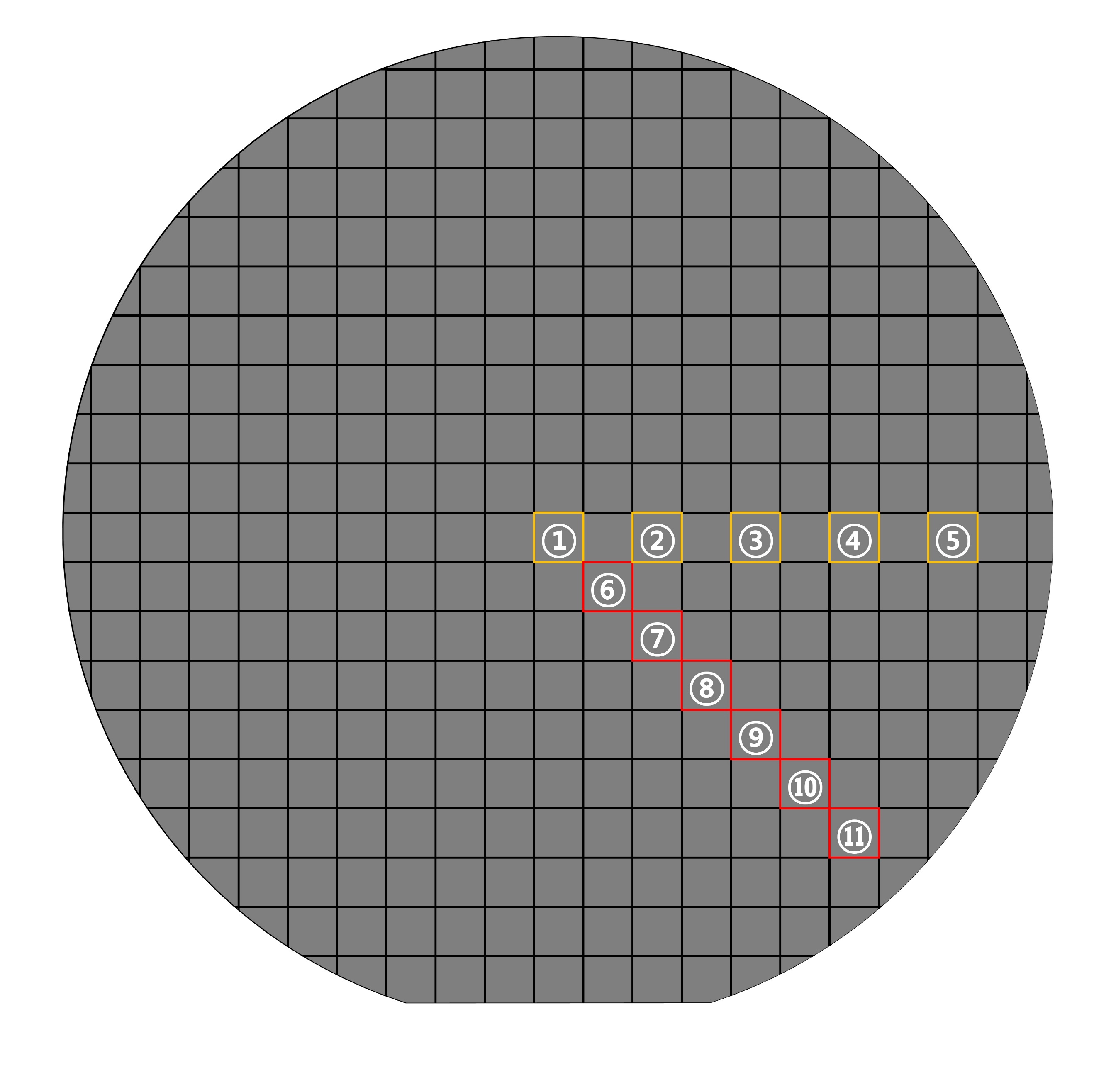
**

**Figure S4:** Schematic of a wafer composed of 313 meta-axicons. The numbers marked on the wafer indicate the positions of the samples used for Depth of Focus (DoF) measurements and correspond to the numbers in Table S2 below.

**Table S2.** Depth of Focus values measured with a photodiode sensor for each sample corresponding to the numbered positions in Figure S4.

| **Sample** | **1** | **2** | **3** | **4** | **5** | **6** | **7** | **8** | **9** | **10** | **11** |
| --- | --- | --- | --- | --- | --- | --- | --- | --- | --- | --- | --- |
| **DoF (mm)** | 2.339 | 2.329 | 2.325 | 2.322 | 2.315 | 2.315 | 2.339 | 2.332 | 2.302 | 2.292 | 2.312 |
